# Evaluation of flavonoids and diverse antioxidant activities of *Sonchus arvensis*

**DOI:** 10.1186/1752-153X-6-126

**Published:** 2012-10-29

**Authors:** Rahmat Ali Khan

**Affiliations:** 1 Department of Biotechnology, Faculty of Biological Sciences, University of Science and technology, Bannu, 2800, KPK, Pakistan

**Keywords:** *Sonchus arvensis*, Correlation, DPPH, Total antioxidant activity, HPLC

## Abstract

**Background:**

*Sonchus arvensis* is used in the treatment of various human aliments as a traditional medicine in Pakistan. In the study its various fractions are characterized for scavenging of diverse free radicals.

**Results:**

Results of the present study revealed that various fractions of *Sonchus arvensis* significantly scavenged the free radicals (DPPH·, ABTS·+, ·OH, superoxide), however its methanolic fraction is more potent than other fractions. Significant correlation was found between DPPH·, ABTS·+, superoxide radical and total antioxidant activity with total flavonoids and phenolics contents. Phytochmical analysis revealed the presence kaempferol, quercetin, orientin, rutin, hyperoside, catechin and myricetin.

**Conclusion:**

From the present data it is concluded that various fractions of *Sonchus arvensis* significantly scavenged the free radical, which might be due the presence of polyphenolic constituent.

## Background

Oxidation provides energy to living organisms for various metabolic processes. Normally a balance is present between amounts of free radicals produced in our body and antiradicals enzymatic and non enzymatic system to quench these and protect the body from their harmful effects [1]. Free radicals including superoxide anions, hydrogen peroxide and hydroxyl are extensively involved in oxidative damage at cellular level. Growing number of evidences suggest that reactive oxygen species (ROS) induced biochemical changes are decisive factors in various chronic human disease such as diabetes mellitus, cancer, atherosclerosis, arthritis, inflammation and neurodegeneration [2]. Human body has developed many mechanisms both enzymatic and nonenzymatic to eliminate ROS but not enough in severe oxidative stress conditions. Many studies have been carried out to investigate, that how to avoid the onset of oxidative diseases. The most studied system to fight against oxidative stress is to combat their level in body by supplying with greater amount of natural antioxidants, which can be attained by elevated use of vegetables and fruits. Natural antioxidants, particularly polyphenolics are safe and also bioactive. For that reason, in recent times, extensive studies have been conducted to identify plants with antiradical aptitude that may be used for human consumption [3]. The plant based therapeutics against oxidative stress induced diseases is the research of medicament of these days. The intake of fresh fruits, vegetables and tea rich in natural antioxidants has been connected with prevention of cancer and cardiovascular diseases [4]. The higher intake of plant foods correlates with lower risk of mortality from these diseases [5]. Approximately 60% of the commercially available anti-tumoral and anti-infective agents are of natural origin [6]. Polyphenols are the most significant constituents of plants for the antioxidant properties. The antiradical property of polyphenols is mostly because of their redox properties, which allow them to perform as reducing agents, singlet oxygen quenchers, metal chelators, hydrogen donors and reductants of ferryl hemoglobin [7-12]. Sonchus species are widely distributed throughout the Pakistan. Their aerial parts, popularly known as ‘kucai’, which remain the cheapest source of protein, vitamins, minerals and essential amino acids in the diet of many people, may be of great importance in helping to alleviate various human ailments such as hepatotoxicity [13], nephrotoxcity [14,15], cardiotoxicity [16], astma [17], brain dysfunction [18], adrenal [19] and oxidative stress [20].

## Result and discussion

### Phytochemical characterization

#### Total phenolics and flavonoids contents

Medicinal plant and their bioactive phenolic and flavonoids content play important role in scavenging of free radicals. Tables 1 and 2 represent the concentration of total phenolics and flavonoids contents in various fractions of *Sonchus arvensis*. *Sonchus arvensis* methanolic fraction showed greater concentration of total phenolics contents (420 ± 6.9) as compare to other fractions viz; chloroform (315 ± 9.3), ethyl acetate (292 ± 3.0) and n-hexane (131 ± 2.3) mg GAE/g respectively. Total flavonoid concentration are varied from 1.3±0.04 to be 7.2±0.03 with a descending order of methanol > chloroform > ethyl acetate > n-hexane fraction. Methanolic fraction showed significantly (*P*<0.01) high amount of total extractable phenolic and flavonoid compounds comparatively to chloroform, ethyl acetate and n-hexane fraction (Tables 1 and 2). Similar results were obtained by Khan *et al.*[21]. 

**Table 1:** **Total flavonoid compounds and extraction yield in various fractions of *Sonchus arvensis***

***Sonchus arvensis *fraction**	**Total flavonoids compounds as rutin equivalent (mg/g dry extract)**	**% yield extraction**
Methanolic extract	23.4 ± 1.2^d^	1.9 ± 0.03^c^
Chloroform fraction	16.7 ± 2.0^c^	1.0 ± 0.04^b^
Ethyl acetate fraction	10.4 ± 1.0^b^	0.8 ± 0.02^b^
N-hexane fraction	5.01 ± 0.2^a^	0.5 ± 0.01^a^

Each value in the table is represented as Mean ± SD (n=3) Means not sharing the same letter are significantly different (LSD) at *P<0.01* probability level in each column.

**Table 2:** **Total phenolic compounds and extraction yield in various fractions of *Sonchus arvensis***

***Sonchus arvensis *fraction**	**Total phenolic compounds as mg gallic acid equivalent (GAE mg/g extract)**	**% yield extraction**
Methanolic extract	420 ± 6.9^c^	7.2 ± 0.04^d^
Chloroform fraction	315 ± 9.3^b^	5.2 ± 0.07^c^
Ethyl acetate fraction	292 ± 3.0^b^	3.2 ± 0.05^b^
N-hexane fraction	131 ± 2.3^a^	1.3 ± 0.03^a^

Each value in the table is represented as Mean ± SD (n=3) Means not sharing the same letter are significantly different (LSD) at P<0.01 probability level in each column.

### HPLC quantification of flavonoids

**Figure 1:**
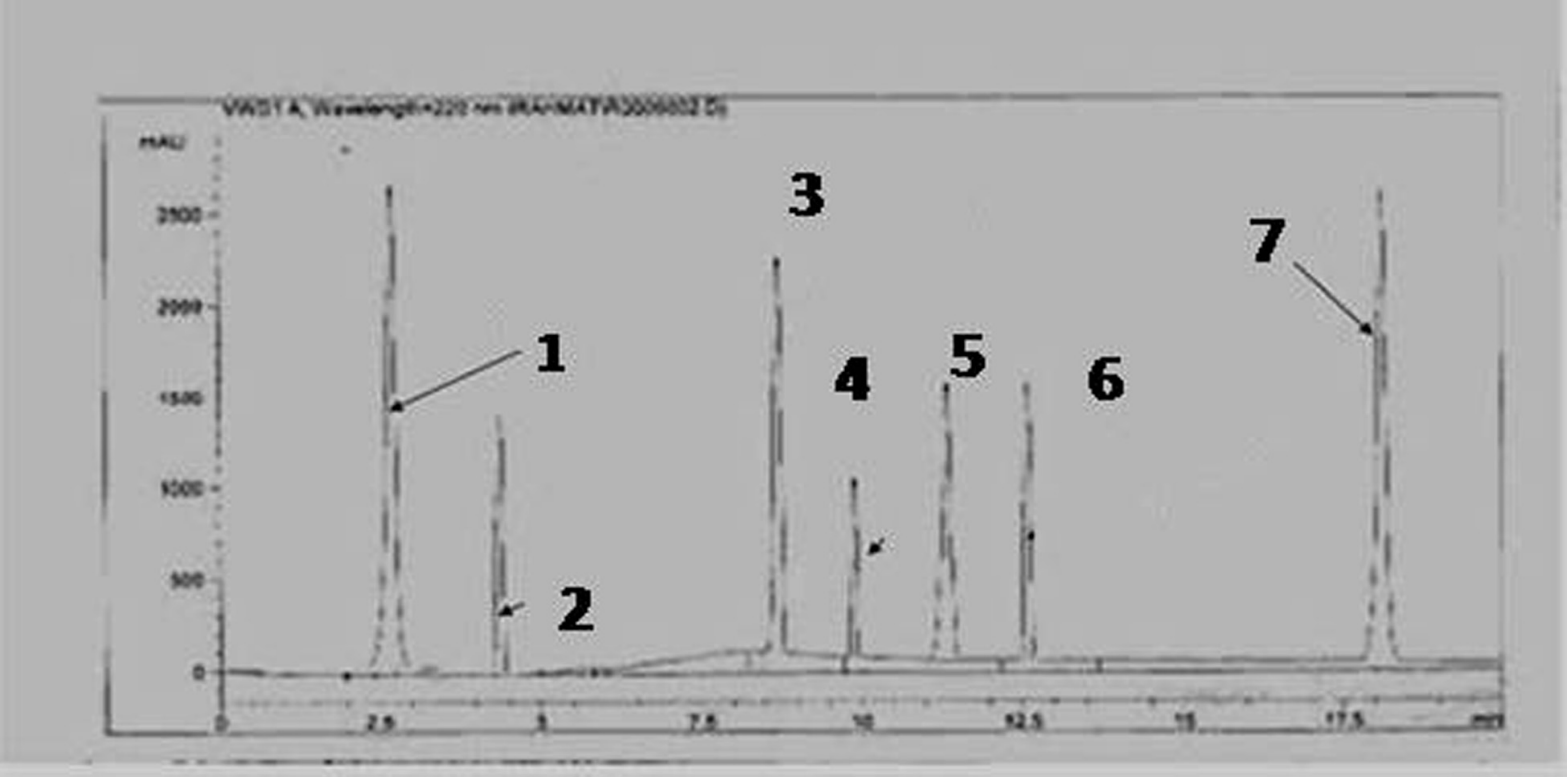
**HPLC fingerprints obtained from *Sonchus arvensis *eluted with mixtures trifluoroacetic acid and acetonitrile indicated the presence of six compounds 1.; (orientin), 2.; (hyperuside), 3.; (quercetin) 4.; (catechin) 5.; (rutin), 6.; (myricetin) and 7; (kaempferol).**

Various food supplements and medicinal plants possess bioactive polyphenolic flavonoids which effectively scavenging the reactive oxygen species because of their phenolics hydroxyl groups [22]. *Sonchus arvensis* powder was characterized for the presence of polyphenolic constituents, revealed the presence of orientin, rutin, kaempferol, myricetin, hyperuside, catechin and quercetin using integration peak-areas at 220 nm for quantification as shown in Figure 1. Various standard compounds were used to obtain calibration curves using least-squares linear regression. The linearity of all calibration curves was determined by calculating the correlation coefficients. Quantification of these compounds is shown in Table 3. Other researcher also reported similar results showing the presence of the bioactive constituent during chemical characterization of medicinal plants [23-26]. 

**Table 3:** **HPLC quantification of *Sonchus arvensis***

**Retention time**	**Concentration (μg/mg dry weight)**	**Compound**
3.5	0.871 ± 0.05	Orientin
4.0	0.455 ± 0.03	Hyperuside
8.0	0.780 ± 0.03	Quercetin
9.8	0.565 ± 0.07	Catechin
11.2	0.457 ± 0.02	Rutin
12.5	0.647 ± 0.07	Myricetin
18.5	0.947 ± 0.1	Kaempferol

Mean±SD.

### Role of various fractions of *Sonchus arvensis* in scavenging of diverse free radicals

Different free radical scavenging methods are used in this study to determine antioxidant efficacy of various *Sonchus arvensis* fractions.

Free radicals of 1, 1-diphenyl 1-2-picrylhydrazyl (DPPH) are widely used for screening of medicinal plants to investigate their antioxidant potential. The principle of this antioxidant assay is the capability of DPPH, a stable free radical, to diminish the color in the presence of antioxidants. The deep purple color of DPPH radical is due to the presence of an odd electron in it [27]. When an electron donated by an antioxidant compound to DPPH, the DPPH is decolorized, this can easily be quantified by noting the change in absorbance at 515 nm. The scavenging effect on DPPH radical was varied significantly by different fractions (Table 4). Oszmianski *et al.*[28] resulted similar antioxidant activities against DPPH. Various fractions of *Sonchus arvensis* revealed considerably different in their ABTS radical cation scavenging activities as shown in Table 4 dependent on solvent polarity might be due the prensence high molecular weight phenolics such as catechin, and rutin derivatives in addition to other flavonoids. Hagerman *et al.*[29] reported similar results in his experiment that the high molecular weight phenolics responsible for quenching of ABTS cation. The superoxide radical (O^-2^) is produced by various biological reactions is very lethal free radical [30]. The hydroxyl radical generates toxic effects by interacting with DNA bases, proteins, and amino acids. The extracts reduce the photochemical reaction of NBT in the presence of riboflavin. NBT reduced into tetrazoinyl radical in the presence of (O^-2^) that can transform to the formazan [30,31]. Results of the present study (Table 4) indicated that methanolic fraction (35 ± 1.7) possess greater scavenging ability as chloroform (45.7 ± 2.2), ethyl acetate (65.3 ± 2.7) and n-hexane fractions (89.2 ± 3.9). 

**Table 4:** **IC**_**50 **_**of various fractions of *Sonchus *for diverse antioxidant systems**

***Sonchus arvensis * fraction**	**Super oxide radical scavenging assay**	**DPPH radical scavenging assay activity**	**ABTS**^**+**^**radical scavenging assay**
Methanolic extract	35 ± 1.7 ^a^	3.4 ± 0.3^a^	65.7 ± 3.7^a^
Chloroform fraction	45.7 ± 2.2 ^b^	4.1 ± 0.5^a^	81.5 ± 4.5^b^
Ethyl acetate fraction	65.3 ± 2.7 ^c^	6.7 ± 0.2^b^	100.7 ± 7.5^c^
N-hexane fraction	89.2 ± 3.9 ^d^	10.1 ± 0.8^c^	123.5 ± 5.8^d^
Ascorbic acid	69.6 ± 4.1 ^c^	3.9 ± 0.7^a^	86.3 ± 4.5^b^

Each value in the table is represented as Mean ± SD (n=3) Means not sharing the same letter are significantly different (LSD) at P<0.01 probability level in each column.

The basic principle to assess the antioxidant capacity through phosphomolybdenum assay includes the reduction of Mo (VI) to Mo (V) by the plant extract possessing antioxidant compounds. In the present study addition of the various fractions of *Sonchus arvensis* showed reduction (Tables 4 and 5), suggested due the presence of effective antioxidants in various fractions as revealed in other study of literature [32]. Hydrogen peroxide non reactive, but sometimes it can be toxic to living cells, because in living cell it is converted into free radical called hydroxyl radicals (^·^OH), react with biomolecules, cause tissue damage and cell death. Various fractions of *Sonchus arvensis* markedly scavenging of (^·^OH) as shown in Table 4. Reduction of the iron ion is an indicator of electron-donating activity, which is an important mechanism of phenolic antioxidant action. Yellow color of test solution changes to various shades of green and blue depending upon the reducing power of each extract. Various fractions of *Sonchus arvensis* showed significant reduction in order methanolic < chloroform < ethyl acetate < n-hexane fraction due to the presence of reductant (antioxidants) which causes the reduction of Fe^3+^/Ferric cyanide complex to ferrous form. 

**Table 5:** **IC**_**50 **_**of various fractions of *Sonchus arvensis *for diverse antioxidant systems**

***Sonchus arvensis* fraction**	**Reducing power**	**Hydroxyl scavenging assay**	**Hydrogen peroxide scavenging assay**	**Total antioxidant activity**
Methanolic extract	53.1 ± 2.7 ^a^	26.4 ± 0.9^a^	86.4 ± 7.1 ^a^	67.0 ± 3.8 ^a^
Chloroform fraction	81.3 ± 3.2 ^b^	38.1 ± 1.6^b^	90.2 ± 8.5 ^b^	90.7 ± 5.9 ^b^
Ethyl acetate fraction	112.4 ± 5.1^c^	43.1 ± 3.9^b^	120.3 ± 9.5 ^b^	110.5 ± 7.0 ^c^
N-hexane fraction	134.6 ± 4.6^d^	78.6 ± 3.2^c^	145.1 ± 6.6 ^c^	1322.21 ± 8.4 ^d^
Ascorbic acid	55.7 ± 2.0 ^a^	24.2 ± 2.65^a^	90.3 ± 7.5 ^a^	70.3 ± 3.5 ^a^

Each value in the table is represented as Mean ± SD (n=3) Means not sharing the same letter are significantly different (LSD) at P<0.01 probability level in each column.

### Correlations of antioxidant activities, phenolics and flavonoids content of *Sonchus arvensis*

Table 6 revealed that there is a significant *(P<0.01)* correlation between DPPH (0.892^b^), reducing power (0.823^b^), superoxide radical (0.670^c^), hydrogen peroxide (0.790^b^) and total antioxidant activity (0.976^a^) and total phenolic contents in various fractions of extract while non significant correlation was observed of hydroxyl (0.303) and ABTS^+^ radical (0.452) with total phenolic contents. Similar correlation is present between flavonoids contents and various free radicals used in this study. Present study also revealed that methanolic fraction is more potent as compared to other fractions [33,34], which have shown that high total polyphenol content increases the antioxidant activity and proves a linear correlation between phenolics content and antioxidant activity. Methanolic fraction exhibited a significant correlation as was reported by Bortolomeazzi *et al.*[35] and Sahreen *et al.*[36]. 

**Table 6:** **Correlations between the IC**_**50 **_**values of antioxidant activities, phenolics and flavonoids content of *Sonchus arvensis***

**Assays (IC**_**50**_**μg/ml)**	**Correlations R**^**2**^	
	**Phenolics**	**Flavonoids**
DPPH	0.892 ^b^	0.981 ^a^
Reducing power	0.823 ^b^	0.821^b^
Super oxide radical scavenging	0.670 ^c^	0.723 ^c^
Hydrogen peroxide scavenging assay	0.790^b^	0.694 ^c^
Hydroxyl radical scavenging activity	0.303	0.206
ABTS^+^ radical scavenging assay	0.452	0.347
Total antioxidant activity	0.976 ^a^	0.712 ^c^

Various fractions of *Sonchus arvensis* were used in the correlation. a, b, c indicate significance at *P<0.05*, *P<0.01* and *P<0.001* respectively.

## Materials and methods

### Chemicals

Nitroblue tetrazolium (NBT), β-nicotinamide adenine dinucleotide reduced (β-NADH), 2-deoxy- D-ribose, linoleic acid, ammonium thiocyanate, β-carotene, 3-(2-pyridyl)-5, 6 bis (4-phenylsulfonic acid)-1,2,4-triazine (ferrozine), Phenazine methosulphate (PMS), 2,2-diphenyl-1-picrylhydrazyl (DPPH), ethylenediamine tetra acetic acid (EDTA), rutin, ascorbic acid, gallic acid, potassium ferricyanide; trichloroacetic acid (TCA), thiobarbituric acids (TBA) were obtained from Sigma Aldrich Chemical Co. (USA). All other reagents were of analytical grade.

### Plant collection

*Sonchus arvensis* was collected from District Bannu (Pakistan) during May 2011 at maturaty. Plants were identified and a voucher specimen (R0023) was submitted at Herbarium of University of Science and Technology Bannu, KPK, Pakistan. Whole plant (leaves, stem, flowers and seeds) were shades dried at room temperature for two weeks, chopped, ground mechanically of mesh size 1 mm.

### Preparation of plant extracts

2 kg powder fine powder was socked in 4 liter of 80% hydroxyl methanol with random shaking for 7 days. After a week the extract was filtered through whatmann filter paper No. 45 and the filtrate was evaporated through rotary vacuum evaporator at 40°C to get methanolic crude extracts (SME). The crude extract was suspended in water and fractionated by liquid: liquid partition with solvents of increasing polarity; starting from n-hexane, ethyl acetate and chloroform. All the fractions were stored at 4°C for further phytochemical and *in vitro* investigations.

### Phytochemical investigation

#### Total phenolic contents

Various fractions of *Sonchus arvensis* were used for determination of total phenolic content (TPC) using Folin-Ciocalteu reagent [37]. 400 μl Folin-Ciocalteu reagents were mixed with 200 μl of various fractions (1.0 mg/ml). The solution was incubated for 5–10 min at 25°C and mixed with 0.2 ml of 7% Na_2_CO_3_ solution and incubated for two h 25°C before taking the absorbance at 725 nm. Gallic acid was used to plat standard calibration curve and total phenolics were calculated as per mg gallic acid (GAE) equivalents per gram of dried fraction (mg/g).

### Total flavonoid content

Total flavonoids content was determined by using the protocol of Sakanaka *et al.*[38]. Briefly, 0.25 ml of each fraction (1 mg/ml) and rutin standard solution (15–250 μg/ml) was mixed with 1.25 ml of distilled water in a test tube, followed by addition of 75 μl of a 5% (w/v) sodium nitrite solution. After 6 min, 150 μl of 10% (w/v) aluminum chloride solution was added, and the mixture was allowed to stand for a further 5 min before 0.5 ml of 1 M NaOH was added. The mixture was made up to 2.5 ml with distilled water and mixed well. The absorbance was measured immediately at 510 nm. The results of samples were expressed as mg of rutin equivalents of total extractable compounds.

### High performance liquid chromatography

For determination bioactive constituents responsible for pharmacological activities 1 g fine powder of *Sonchus arvensis* was extracted with 25% hydrochloric acid and methanol for 1 h. The extract was diluted with methanol. 10 μl samples were injected into the HPLC apparatus. Separation was carried out through column (5 μm; 4.6×150 mm, Agilent) with UV–vis detector. Solvent A (0.05% trifluoroacetic acid) and solvent B (0.038% trifluoroacetic acid in 83% acetonitrile (v/v) with the following gradient: 0–5 min, 15% B in A, 5–10 min, 70% B in A, 10–15 min, 70% B in A are used for separation. The flow rate was 1 ml/min and injection volume was 10 μl. Six different standards compounds (myricetin, catechin, kaempferol, quercetin orientin, hyperuside, and rutin) were run for comparable detection and optimized. The calibration curves were defined for each compound in the range of sample quantity 0.02–0.5 μg. All samples were assayed in triplicate. All quantitative data were explained by analyst software.

### Pharmacological assessment

Various fractions of *Sonchus arvensis* were characterized for scavenging of various free radicals. All these methods are expressed with IC50.

#### Scavenging of diphenylpicrylhydrazyl (DPPH) radical

DPPH free radical scvanging activity of various fractions of *Sonchus arvensis* was achived using the standard protocol of Gyamfi *et al.*[39]. A stock solution of DPPH was made by dissolving 2.4 mg DPPH in 100 ml of methanol and diluted after achieving an absorbance of 0.980 (± 0.02) at 517nm. 500 μl of DPPH solution was mixed with 500 μl of each fraction (50–250 μg/ml) and incubated for 30 min in dark after vigorous shacking. Absorbance was recorded at 517 nm and DPPH scavenging activity of various fractions was calculated by the following equation: Percentage Inhibition (%) = [(Absorbance of control-Absorbance of sample) / (Absorbance of control)] × 100. IC_50_ values obtained as to determine the 50% inhibition of DPPH radicals. Ascorbic acid was used as standards.

#### ABTS radical scavenging

To assess the ABTS free radical scavenging stock solution was prepared by mixing equal volumes of 7 mM ABTS solution and 2.45 mM potassium persulfate solution [40]. The mixture was incubated at room temperature in the dark for 12 h to yield a dark-colored solution containing ABTS^·+^ radicals and diluted for an initial absorbance of about 0.700 (±0.02) at 745 nm. 300 μl of each fraction (25–250 μg/ml) was mixed with 2.7 ml of ABTS working solution and measured absorbance exactly 1 min after mixing the solution. The final absorbance was noted up to 6 min and data for each assay was recorded in triplicate. Ascorbic acid was used as positive controls. The scavenging activity was estimated based on the percentage of ABTS radicals scavenged by the following formula: % scavenging = (A_0_ – A_S_ / A_0_) × 100, Where A_0_ is absorption of control, A_S_ is absorption of tested extract solution.

#### Superoxide radical scavenging

The riboflavin-light-NBT system was employed to assay the superoxide radical [31]. The reaction mixture consisted of 500 μl of 50 mM phosphate buffer (pH 7.6), 300 μl of 50 mM riboflavin, 250 μl of 20 mM PMS and 100 μl of 0.5 mM NBT before adding up of 1.0 ml of various fractions. Reaction was initiated by illuminating the above solution using a fluorescent lamp. After placing there for 20 min, the absorbance was recorded at 560 nm. The percentage of scavenging superoxide anion generation was calculated as: Percentage inhibition (%) = (1- sample absorbance/control absorbance) × 100.

#### Total antioxidant capacity

Reduction of phosphomolybdenum was calculated to determine the total antioxidant capacity of different samples by adapting the method described by Prieto *et al.*[41]. 100 μl of each fraction was mixed with the reagent solution (1.0 ml) consisting of phosphate buffer, 0.6 M H_2_SO_4_, 28.0 mM sodium molybdate and 4.0 mM ammonium molybdate. The mixture was placed in a water bath at 95°C for 90 min. After cooling to room temperature, the absorbance was taken at 765 nm. A standard of ascorbic acid was employed. Total antioxidant scavenging capacity was calculated as: Total Antioxidant capacity (%) = [(Abs. of control-Abs. of sample) / (Abs. of control] ×100.

#### Hydrogen peroxide scavenging

The scavenging capacity for hydrogen peroxide was measured according to the method [42]. A solution of hydrogen peroxide (2 mM) was prepared in 50 mM phosphate buffer (pH 7.4). Hydrogen peroxide concentration was determined spectrophotometrically at 230 nm absorption using the molar extinction coefficient for H_2_O_2_ of 81 mol^-1^cm^-1^. 0.1 ml of various fractions (25–250 μg/ml in respective solvents), ascorbic acid was transferred into the test tubes and their volumes were made up to 0.4 ml with 50 mM phosphate buffer (pH 7.4) or solvent (methanol). After addition of 0.6 ml hydrogen peroxide solution, tubes were vortexed and absorbance of the hydrogen peroxide at 230 nm was determined after 10 min, against a blank. 50 mM phosphate buffer without hydrogen peroxide was used as blank. Hydrogen peroxide scavenging ability (in triplicate) was calculated by the formula:% scavenging = (1–Ae/Ao) × 100, where Ao is the absorbance without sample, and Ae is absorbance with sample.

#### Hydroxyl radical scavenging

The effect of extracts on hydroxyl radicals was assayed by using the deoxyribose method [43]. 2-Deoxyribose is degraded on exposure to hydroxyl radicals generated by Fenton's reaction. Each fraction and ascorbic acid (ASA) was prepared in methanol. The reaction mixture contained 450 μl of 0.2 M sodium phosphate buffer (pH 7.0), 150 μl of 10mM 2-deoxyribose, 150 μl of 10 mM FeSO_4_-EDTA, 150 μl of 10 mM H_2_O_2_, 525 μl of H_2_O, and 75 μl of sample solution (0.050–0.250 mg/ml in respective solvents). The reaction was started by the addition of H_2_O_2_. After incubation at 37°C for 4 h, the reaction was stopped by adding 750 μl of 2.8% trichloroacetic acid and 750 μl of 1% TBA in 50 mM NaOH, the solution was boiled for 10 min, and then cooled in water. The absorbance of the solution was measured at 520 nm. Ascorbic acid (0.05–0.250 mg/ml) was used as a positive control. The ability to scavenge the hydroxyl radical was calculated using the following equation: % Hydroxyl radicals scavenging activity = (1– absorbance of sample / absorbance of control) × 100.

#### Reducing power

The ability to reduce Fe (III) to Fe (II) was obtained by the procedure stated [44] with some modifications. Extract solution of 1 ml was mixed with 2.5 ml of 0.2 M phosphate buffer (pH 6.6) and 2.5 ml of 1% potassium ferricyanide and then mixture was placed at 50°C for 20 min. 2.5 ml of 10% TCA was added to the solution and centrifuged at 3000 rpm for 10 min. A 2.5 ml of supernatant was mixed with 2.5 ml of distilled water and 500 μl of 1% FeCl_3_ and absorbance was noted at 700 nm. Increase in reducing power of extracts was deduced by an increase in absorbance. Ascorbic acid was used as standard in this experiment.

#### Statistical analysis

Readings for all scavenging assays were taken in triplicate. Graph Pad Prism 5 software was used to calculate the IC50 values. Standard deviation and ANOVA was employed to investigate the differences among IC50 of different fractions for different antiradical assays. Pearson correlation coefficient for phenolic and flavonoids was also employed. All the assays findings were subjected to Student’s t test (P < 0.05; P < 0.01) to find their significance.

## Conclusion

Various fractions of *Sonchus arvensis* revealed significant afficecy in scavenging of free radicals due the presence of poly phenolics compounds as reported via HPLC. The extract can be utilized as an effective and safe antioxidant source, as ethnomedicine and on a commercial basis for the development of drugs.

## Competing interests

The authors declare that they have no competing interests.

## Authors’ contributions

RAK made significant contribution to acquisition of data, analysis, drafting of the manuscript, conception and design. Author read and approved the final manuscript.
